# Structural switching of tubulin in the microtubule lattice

**DOI:** 10.1042/BST20240360

**Published:** 2025-02-05

**Authors:** Yean-Ming Chew, Robert A. Cross

**Affiliations:** 1 Centre for Mechanochemical Cell Biology, University of Warwick, Warwick Medical School, Coventry CV4 7LA, U.K.

## Abstract

Microtubule (MT) dynamic instability, a cycle of growth, catastrophe, shrinkage and rescue, is driven by the switching of tubulin between two structural states, one stabilised by GTP and the other by GDP. Recent work has uncovered the ancient origins of this structural switch and revealed further fundamental elements of microtubule dynamic instability, whereby switching can be brought about by a range of allosteric effectors, propagate deep within the lattice of assembled MTs, and profoundly affect MT function. Here, we review evidence for structural switching within the MT lattice and discuss current ideas about its mechanisms.

## Introduction

Microtubule (MT) dynamic instability is fundamental to the self-organisation of eukaryotic cells. Dynamic instability is driven by a cycle of GTP turnover that switches individual tubulin heterodimers between a lattice-friendly state with bound GTP and a lattice-unfriendly state with bound GDP. The GTP binding site is exposed to solvent at the plus end of the MT [1]. As assembly proceeds, newly arriving heterodimers donate residues that complete the active sites of the underlying subunits, triggering them to hydrolyse their GTP. Phosphate release follows, reverting the underlying subunits to their lattice-unfriendly GDP conformation. Loss of the overlying cap of lattice-friendly GTP-tubulins triggers rapid disassembly of the underlying GDP-tubulin core of the MT. In this way, GTP turnover drives dynamic instability, enabling rapid re-organisation of the MT network and allowing it to do work in the cell.

Until recently, despite appreciable evidence to the contrary [2], GDP-tubulin heterodimers in solution have been widely thought to have a bent conformation and GTP-tubulin heterodimers to have a straight conformation that fits more readily and stably into the lattice. Recent cryoEM data call this attractively simple picture into question by revealing that GTP-tubulin and GDP-tubulin heterodimers in solution have essentially identical structures [3,4]. Notwithstanding, it is clear that different nucleotides can profoundly affect the structure of tubulin molecules once they are assembled into a MT; for example, it is clear that GMPCPP, a nonhydrolyzable analogue, expands the subunits of brain MTs by about 2% in the axial direction [5], compared to GDP-MTs.

To reconcile these data, we need a model in which the MT lattice catalyses its own growth by recruiting tubulin into a lattice-compatible conformation. This type of model (a ‘lattice model’) has been previously proposed but has so far failed to win the day. Recently, however, leveraging both cryoEM and crystallographic data, including data from ancestral forms of tubulin (and actin), it was proposed that the ability to switch between two fundamentally different conformations is ancient, is conserved and operates at the protofilament (PF) level, at the point of incorporation of individual subunits into a single PF, and not before [4]. In other words, a lattice model is proposed to operate even at the level of individual PFs. How might this work?

### PF-level conformational switching

**Figure 1:**
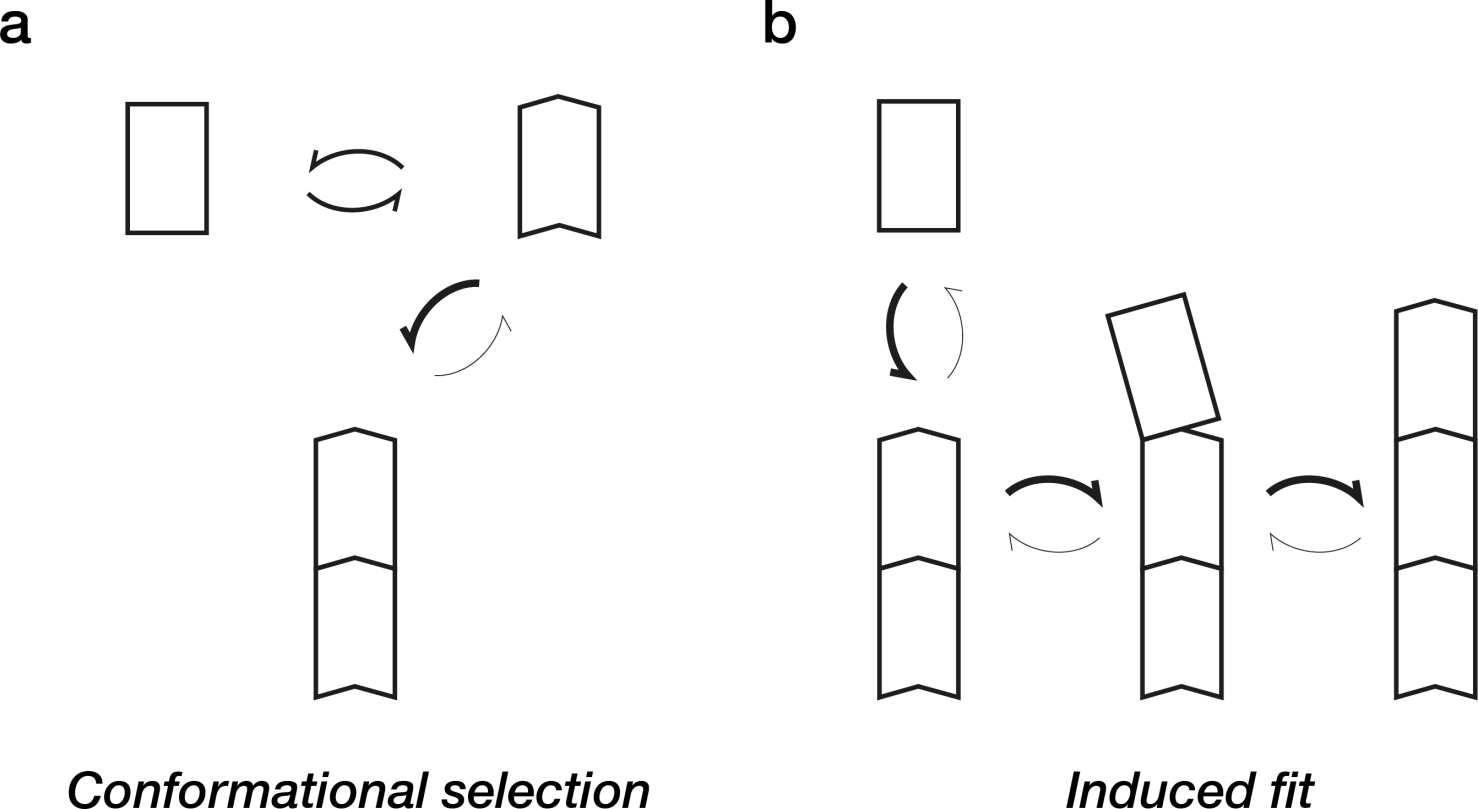
Polymerisation-coupled structural switching. **(a)** In conformational selection, subunits in solution equilibrate between polymerisation-compatible and polymerisation-incompatible conformations. The polymer grows by recruiting only those subunits in the polymerisation-competent conformation. (**b)** By contrast, in an induced fit mechanism, the assembled polymer catalyses conversion of newly arrived subunits into a polymerisation-competent conformation.

In principle, macromolecules can recognise and bind to one another in two different ways. In *conformational selection*, the incoming molecule adopts a conformation that recognises the partner before it binds to the partner. The binding site on the partner selects this conformation. The other possibility is an *induced fit* mechanism, in which the incoming subunit anneals to its partner in a series of smaller steps, shifting progressively into a fully binding-compatible conformation (Figure 1).

Of course, a blend of both mechanisms is possible, but for tubulins (and actins), the evidence points persuasively to an induced fit mechanism, in which access to a polymerisation-compatible conformation is accelerated (catalysed) by the polymer [6]. The recent work [4] argues that this type of induced fit mechanism operates at the level of individual PFs.

The principle underlying this behaviour is that the structural switch of each newly arrived subunit is operated by its neighbours in the PF – predominantly by its underlying neighbour, but also potentially by its overlying neighbour. Armed with this idea, we can now ask what happens to this switching mechanism when PFs zip to one another laterally to form MTs. How is the ancient and fundamental property of two-state structural switching at the level of an individual PF changed by these additional lateral interactions? To what extent does the formation of lateral inter-PF bonds provide additional opportunities for cells to modulate structural switching? What new structural-mechanical properties can emerge, and what biological value might these have?

### Lattice-level conformational switching

**Figure 2:**
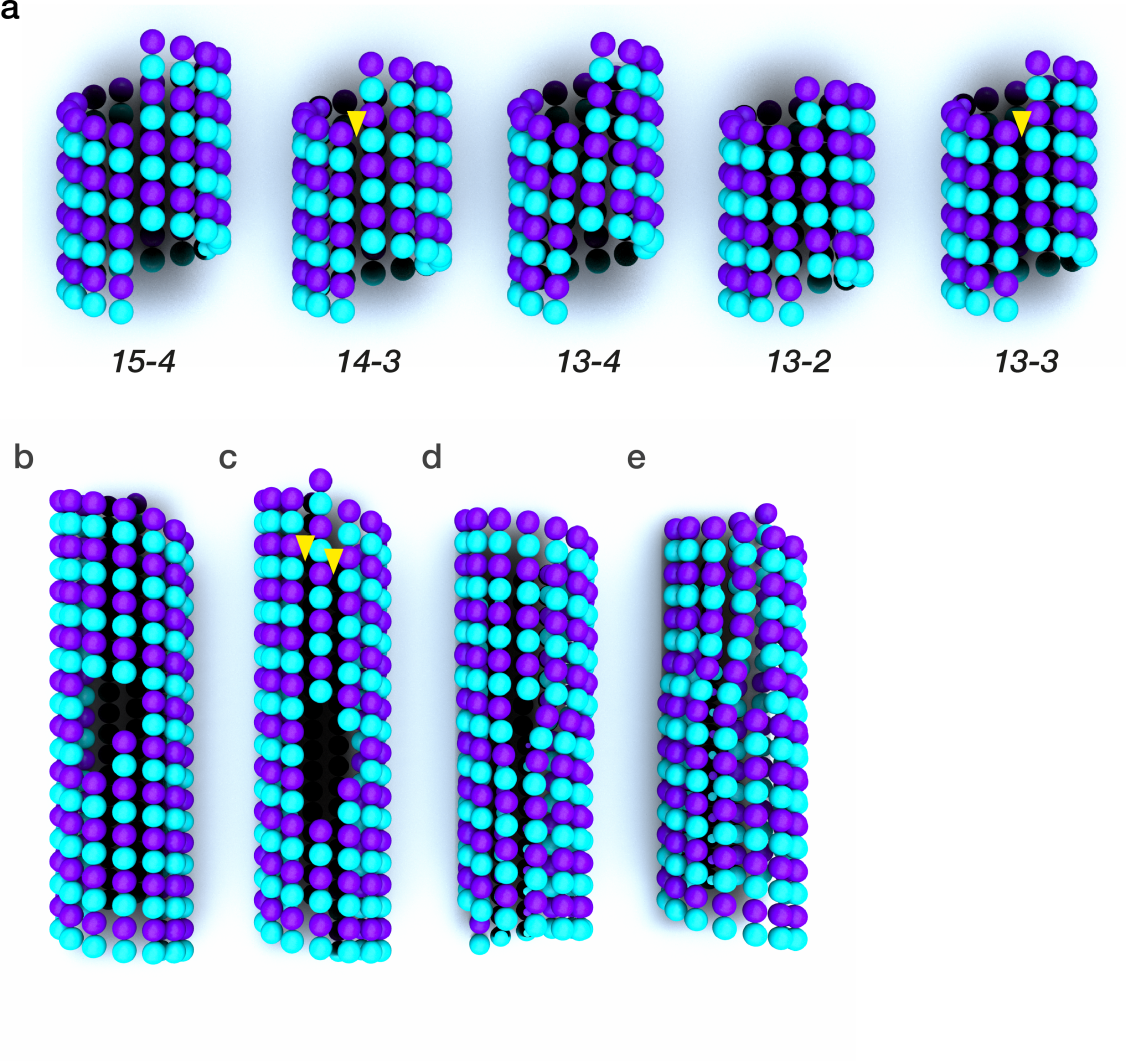
Microtubule architecture is adjustable. **(a)** Microtubules with different PF numbers and helix start numbers. Plus ends, exposing beta tubulin, are at the top. Allosteric effectors can remodel the inter-PF links, causing variations in PF number. The axial staggers between neighbouring PFs that define the A- and B-lattices remain almost unchanged. b-e| Defects of various kinds can disrupt the lattice [7]. **(b)** a vacancy, with no other changes to the lattice. **(c)** a dislocation that adds 2 new A-lattice seams (yellow arrows). **(d)** a vacancy that closes by incorporating a new PF, perhaps by incorporation of a pre-built PF from solution, or perhaps by nucleation of a new PF in situ. **(e)** reduces the PF number by 1. Defects are relatively rare, on the order of 1 per micron in vitro, and 0.1 per micron in vivo [7], but biologically significant [8], because they can exchange tubulin something like MT tips [9].

PFs in natural MTs usually associate side-by-side, in parallel, with a stagger of either 0.9 nm (the B-lattice) or 4.9 nm (the A-lattice). The canonical arrangement is a B-lattice tube of 13 PFs, with a single A-lattice seam (Figure 2a). The patterning of the lateral inter-PF links defines lattice architecture, which can vary appreciably in different natural contexts [10] as well as *in vitro*, and can constrain the actions of allosteric tubulin binders (*below*). Figure 2 shows some architectural variants. A good way to visualise lattice architecture is to imagine taking a sheet of B-lattice PFs, rolling it into a tube, and then joining the edges together so as to include a different number of PFs in the tube [11]. Depending on the PF number, shearing the join optionally introduces an A-lattice seam and/or tilts the PF axes [12] (Figure 2a).

The principle that the MT lattice acts as an allosteric effector for incoming tubulins emerges clearly from *in vitro* reconstitution work [13,14], cell biology [15], and atomistic simulations [16]. PFs form a lattice by establishing lateral connections, in which the M-loop of each tubulin subunit engages with the H1-S2 and H2-S3 loops of its lateral neighbour, in a lock-and-key configuration. The M-loops undergo a disorder-to-order transition as they engage [17]. Their preferred contact angles specify a preferred PF number [18] for the MT. Once established however, the inter-PF links can deform appreciably without breaking, allowing the tube to articulate [19] and the helix start number, the seam number, the PF skew and the PF number all to vary, between and even within individual MTs [7,20] (Figure 2b). All lateral conformational signalling in the lattice needs to pass via the M-loops.

### Guanosine nucleotides as allosteric effectors

**Figure 3:**
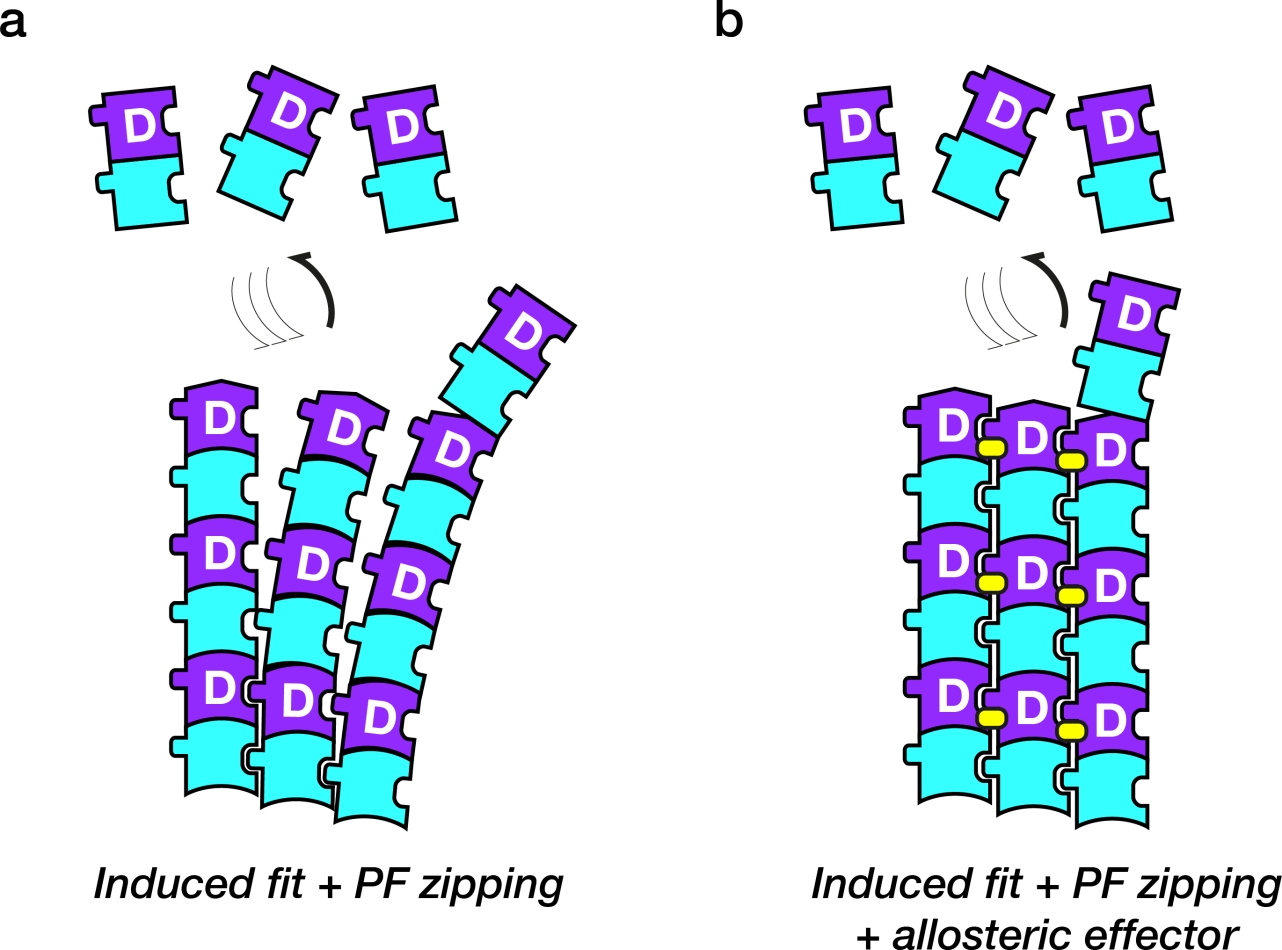
Structural switching linked to lattice assembly. **(a)** Very high free subunit concentrations of GDP-tubulin heterodimers can allow their polymerisation into MTs, directly demonstrating lattice-catalysed switching of GDP-subunits into a lattice-compatible conformation. Zipping together of PFs helps to stabilise tubulin heterodimer subunits in their lattice-compatible conformation. **(b)** Binding of a suitable allosteric effector (yellow symbols) can counteract strain and further stabilise the assembled subunits in their lattice-compatible conformation.

GTP is the prototypical allosteric effector for tubulin. GTP turnover is essential to drive dynamic instability, which is critical for the self-organisation of eukaryotic cells [21]. Thus, the belief that GTP is required to activate tubulin heterodimers for polymerisation appears reasonable. However, it is wrong. Recent work confirms that at concentrations of ~120 µM, GDP-tubulin can polymerise into MTs [22], via minus end growth. This work demonstrates directly that MT growth, like PF growth, uses an induced fit mechanism, where the lattice recruits tubulin into a lattice-compatible conformation (Figure 3). Models with polymerisation-coupled structural switching (Figure 1) predict this behaviour and differ radically from the classical picture of dynamic instability [23,24] as GTP is no longer absolutely required to activate tubulin for assembly. Instead, GTP acts as an allosteric effector that biases polymerisation-coupled structural switching in favour of assembly. Equipped with this idea, it becomes easier to see how lattice binding by many other allosteric effectors can also operate on this putatively ancient and ubiquitous polymerisation-coupled structural switch, thereby affecting not only the exchange equilibria for polymerisation but also conformational switching deep within the lattice.

The binding of GTP [1] to β**-**tubulin deploys its T5 loop [17], whereas the binding of GDP does not. Flipping out the T5 loop displays β**-**tubulin D177, which is thought to interact electrostatically with the α-tubulin of an incoming heterodimer [17]. The incoming subunit supplies residues (α**-**tubulin D251, E254) that complete the β**-**tubulin active site, rendering it competent to hydrolyse MgGTP. The detailed hydrolysis mechanism is not understood [25]. These active site motions couple to global motions of the β-tubulin molecule, chiefly a rotation of the C-term domain and a downward motion of H7 [4]. A point mutant, T238A, at the base of H7, partially suppresses this coupling, producing MTs that grow more processively and shrink more slowly than wild-type [26].

On the basis that GMPCPP MTs have an expanded lattice [5], it was proposed that the stabilising GTP-tubulin cap of dynamic MTs has an expanded lattice [27], while the GDP core is compacted. Recent work shows that things are more subtle [28]. GMPCPP expands, GMPPCP does not, indicating that expansion is due to the un-ATP-like methylene between the α and β phosphates in GMPCPP. Further, in two α**-**tubulin mutants that increase the size of the GTP-cap of dynamic MTs [29], the tip-lattices are not only compacted, they also show opposite skew [30]. Still further, GTPγS lattices are compacted [27,31]. And further yet, in both *Saccharomyces cerevisae* and *Schizosaccharomyces pombe* MTs, both the GTP- and GDP-lattices are expanded – despite yeast MTs showing robust dynamic instability [32,33]. These data suggest that while GTP/GDP-driven structural switching reliably modulates lattice strain, this is not always accompanied by a discernible change in the lattice spacing.

The formation of M-loop links straightens PFs and their subunits, stimulating GTP hydrolysis [34]. Recent cryoEM work [3] firmly supports models in which PFs zip together progressively in an induced fit mechanism. Unzipping of GDP-MTs may also be progressive, albeit faster. GDP lattices have a more tenuous M-loop density [35].

Ayukawa [36] and colleagues revealed details of the earliest stages of GTP-driven MT assembly, using time-resolved electron microscopy of Y222F, a β-tubulin mutant that allows nucleation at lower-than-normal tubulin concentrations. GTP-PFs were found to be straighter and slightly longer than GDP-PFs. GTP shifted the equilibrium between these two and favoured PF zipping, consistent with polymerisation-coupled structural switching.

Current studies aim to determine the conformation of individual molecules within the MT tip lattice [37]. Determining the exact mechanisms by which G-nucleotides influence lattice structure remains a central challenge for the field [1,38].

### Tubulins as allosteric effectors

Complex eukaryotes typically express a large array of tubulin isotypes (humans have 9 α-tubulins and 10 β-tubulins [39], some of which differ from one another by only a few residues. Yet, this is sufficient to profoundly influence function – for example, human single isoform GMPCPP and GDP-taxol α1Bβ2B and α1Bβ3 MTs *in vitro* adopt different PF numbers and exhibit different MT dynamics [18]. Human α1β3 and α1β4 MTs have very different properties [14].

Sequence differences between tubulin isotypes tend to cluster in the C-terminal tails, which can be heavily post-translationally modified [40], but they also cluster in the lateral (M-loop) contact sites. Sequence variations in this region profoundly influence MT architecture and dynamics [35] (Figure 2). For example in *Caenorhabditis elegans*, mec-7 (a β-tubulin) is essential for the formation of 15-PF MT specific to the touch receptor neuron [41]. MTs with different PF numbers grow at characteristically different rates [42]. Shrinkage rates may also be different for different PF numbers. Both single isotype and mosaic-isotype lattices tend to shrink in phases [14,43], and this is suggested to correspond to regions with different PF numbers, separated by defects [14], though this has yet to be directly demonstrated.

Many, perhaps most, natural MTs are mosaics of different isotypes. The mix of isotypes feeds back on lattice architecture and structural switching, which in turn biases the recruitment of effectors such as MT-associated proteins (MAPs) and motors (below). We do not know how the isotype mix in the lattice is achieved – whether the mix of isotypes in the lattice faithfully reflects the isotype mix of free subunits arriving at growing MT tips or whether recruitment is biased. It is possible that certain isotypes are more prone to defect formation, or that certain combinations of isotypes tend to generate defects, but, in general, co-polymerisation of different isotypes, for example, of yeast with brain, seems unproblematic – again reflecting the plasticity of M-loop links. Perhaps relatedly, pure single isoform MTs may also adopt different architectures. For example, α1Aβ3 tubulin with a single E254A point mutation in α1A-tubulin is GTP-hydrolysis deficient and can assemble into MTs with either a 3-start negative skew or a 4-start positive PF skew [30]. This might relate to a tendency toward bistability of the M-loop contact angles [44].

What is clear is that particular tubulin isotypes in the lattice differentially recruit and/or respond to lattice-binding allosteric effectors. Among allosteric effectors, tubulin itself is perhaps the least well understood.

### Motors as allosteric effectors

Some motor proteins can not only recognise but also regulate MT lattice conformation. A clear example is the kinesin-13 subfamily, whose members are non-motile depolymerases that recognise features of the tip-lattice and chaperone tubulin into a curved, lattice-incompatible conformation [45–47]. Kinesin-8 can conform itself to recognise either straight or curved PFs [48], shifting between translocase and depolymerase modes.

**Figure 4:**
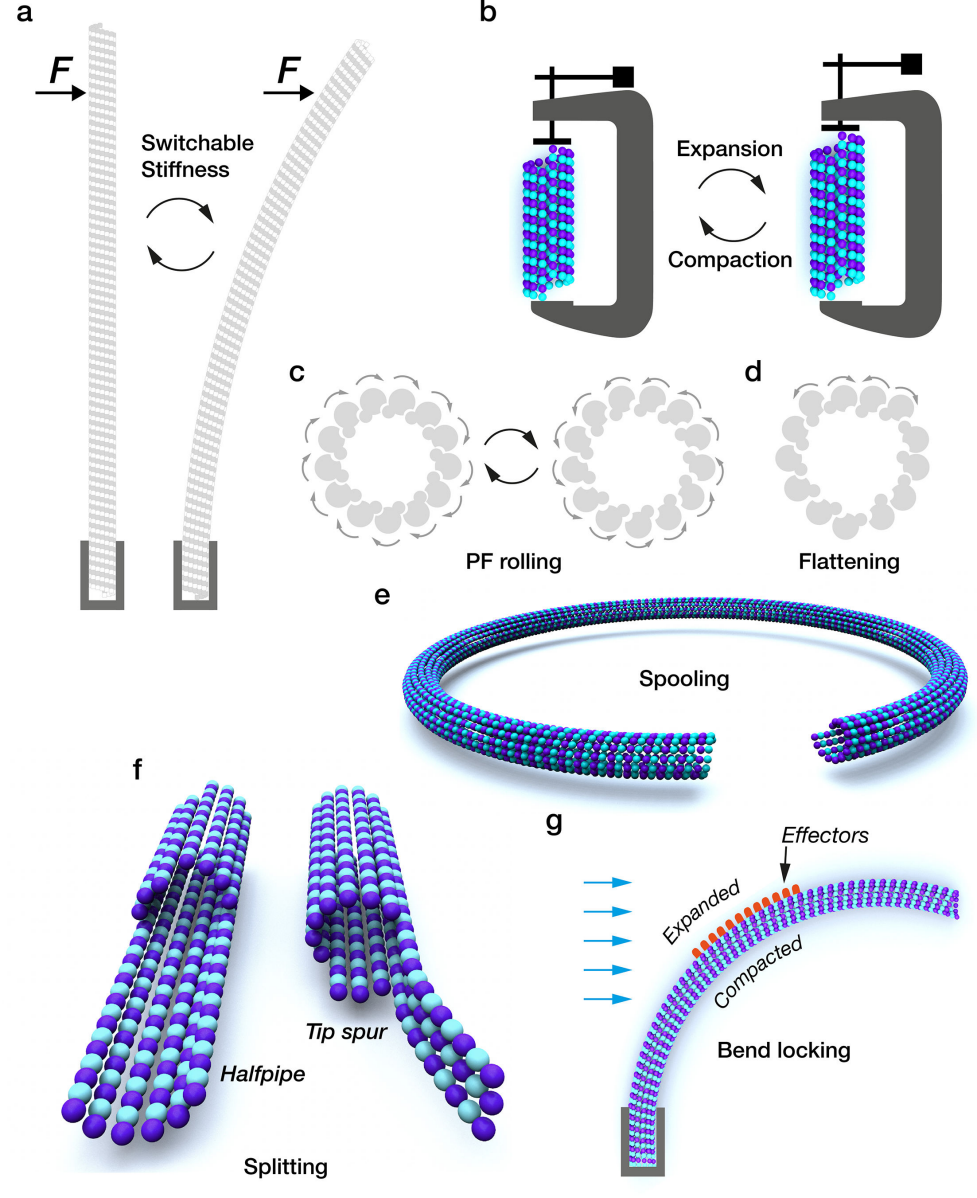
Effector-dependent structural switching in MTs. **(a)** Stiffness changes. (**b)** Effector-driven lattice expansion. (**c)** Shifts in inter-PF roll-angle. (**d)** Concerted local flattening of the lattice. (**e)** MT bends into a ring when gliding on a motor surface. MT spools can have multiple gyres. (**f)** Splitting of the lattice at its tip. Effector-induced expansion of one side of the lattice only can split it apart from the remainder, which depolymerises, creating a halfpipe. Tip spurs can drive spooling as they engage with a motor surface. (**g)** Bending under load in microfluidic flow (blue arrows) expands the lattice on one side and compacts it on the other. Effectors (red dots) can then bind preferentially to the expanded lattice, locking the bend.

The binding of several motile kinesins, including kinesin-1, in their apo state can expand and stabilise the GDP-lattice of mosaic-isotype brain MTs (Figure 4b). These kinesins bind selectively to expanded lattices and move faster on expanded lattices [49,50]. StableMARK, a rigor kinesin construct, selectively labels stable (expanded) MTs in cells [51]. Rigor binding of MTs to a kinesin-1 surface can split the lattice by expanding only those PFs in contact with the kinesin, but not the remainder. Depolymerisation of the unaffected overlying PFs creates a halfpipe [49]. This result suggests that effector-induced lateral allosteric signalling may not extend much beyond nearest neighbours. Gliding of taxol-stabilised MTs on a kinesin surface can split the lattice via creation of curved tip spurs (Figure 4f), which drag the MT sideways, causing it to circle around. This process can create spools with multiple gyres. Curved taxol-stabilised tip spurs that break away continue to orbit in tight circles until they depolymerise [52]. The mechanism of motor-driven splitting and fraying of the minus-end tip lattice remains unclear. Walking kinesin-1 can extract tubulin from defects [53]. This can cause mechanical failure and explosive disassembly [54]. It is not yet clear how motors extract tubulin from the lattice and what proportion of motor-driven tubulin exchange occurs at defects [55].

By bending tethered dynamic MTs in a microfluidic flow and introducing kinesin into the flow, the GDP-lattice can be bend-locked in a curved conformation (Figure 4g). Releasing the kinesin causes the MT to recoil into a straight conformation. This effect might be due to the preferential binding of kinesin to the mechanically expanded exterior (convex) outer surface of the bent MT [49], but this remains to be directly demonstrated. Relatedly, for a few seconds after tethered MTs in a flow are first exposed to kinesin, local expansion occurs, producing multiple kinks in the lattice. As more kinesin arrives, these kinks resolve, and the lattice straightens out into a fully expanded state. This suggests that local lattice expansion may favour kinesin binding in a cooperative feedback loop. Patchy binding of certain kinesins to MTs has been previously noted [56,57]. There is evidence that kinesin-induced expansion can persist after kinesin dissociates. Examples of such hysteresis have been reported, whereby the expanded lattice persists for a minute or so after the motor has dissociated [50,58], though in other situations, motor dissociation returns the lattice to rest length within a second or so [49].

Motor binding can also shift the inter-PF contact angle in the lattice without necessarily influencing PF number (Figure 4c). The DNAH7 domain of axonemal dynein flattens the MT cross-section (Figure 4d). DNAH7 has a higher affinity for such regions of lower curvature [59]. Cross-sectional flattening of MTs under bending load makes them less stiff [60]. Neighbouring PFs in brain MTs tend to counter-rotate (Figure 4d), driven by a tendency for their inter-PF (M-loop) hinge angles to flip between preferred values [44].

An intriguing but so far undemonstrated possibility is that MTs might use motor-driven conformational switching of the lattice to influence dynamic instability, such that MT tips experiencing more traffic would persist in their growth phase for longer. Traffic-dependent effects on the rescue of catastrophes at defect sites have been detected [61].

### MAPS as allosteric effectors

In addition to tubulin isotypes, MT conformations can be regulated by MAPs. Tubulin mutations that influence MAP binding can cause severe human diseases [62].

Some MAPs can modulate lattice expansion and specify PF number by resetting PF contact angles. Doublecortin strengthens the tip-lattice [63] by binding in the groove between PFs at the junction of four heterodimers. It restricts PF number to 13 [64]. End-binding (EB) proteins bind tightly to MT tips and occupy the inter-PF groove, again driving towards 13 PFs. EB binding can axially compact the lattice and convert positively skewed E254N GTP-hydrolysis-deficient mutant MTs to a negative skew conformation, which favours EB binding [28].

TOG polymerases (MCAK) are MAPs that configure tubulin into a semi-lattice-compatible configuration. At high tubulin concentrations, they promote assembly, while at low tubulin concentrations, they promote disassembly. CLASPs, a subclass of TOG-based MAPs, stabilise a pausing conformation, holding tubulin at a tipping point slightly favouring disassembly [65].

Katanin and spastin, AAA ATPase mechanoenzymes, extract tubulin from the lattice, thereby creating defects (Figure 2) that show increased tubulin exchange. When supplied with GTP-tubulin, katanin can inject GTP-tubulin into the lattice, creating an island that promotes the rescue of shrinking MTs [66].

At high concentrations, certain MAPS undergo phase separation. Tau envelopes, formed of phase-separated tau, can reverse the taxol-induced expansion of brain MTs [67], with potential relevance to Tauopathies involving tau overexpression. Evidence for a physiological role for phase separation of MAPs in general is ‘often lacking’ [68].

### Drugs (MTAs) as allosteric effectors

MT-targeting agents (MTAs) [69] bind at seven different tubulin sites [70] and profoundly alter their function. Taxol, the archetype, illustrates the power of MTAs as chemical tools to control structural switching and reshape lattice structure–function. Taxol binds abutting the β-tubulin M-loop and, through allosteric effects at this site, can assemble tubulin without the addition of exogenous GTP [71]. Taxol also binds to isolated PFs and tends to straighten them [72], albeit curved PF tip spurs developed in kinesin gliding assays are also stabilised by taxol (Figure 4f). Recent data clarify that taxol binds four orders of magnitude more weakly to free tubulin in solution than to tubulin polymerised into MTs [29]. It seems clear now that taxol only binds tightly once tubulin has been manipulated by the lattice [29]. Perhaps relatedly, taxol can have different structural effects when added before assembly compared to after [73]. Taxol can also influence lattice stiffness [74].

Once bound, taxol acts as a conformational wedge, biasing against reversion to the lattice-unfriendly, compacted, GDP-tubulin conformation. Taxol derivatives clarify that lattice expansion and stabilisation are separable, albeit typically coupled; baccatin III expands the GDP-lattice without stabilising it [29]. FChitax3 shifts the PF number of brain MTs towards 15 or 16. FChitax3 can create defects. It binds preferentially to defects and tips. Its effect on PF number changes the MT growth rate^[75]^.

### Mechanical force as an allosteric effector

Mechanical force in cytoskeletal systems can act in a manner similar to a chemical kinetic inhibitor [76] or an allosteric effector. Bend locking [49] (Figure 4g) strongly suggests that mechanical forces exerted on the MT can influence effector binding. Effector-induced lattice expansion can recruit and accelerate kinesin [49,50]. Directly stretching MTs with mechanical force can accelerate dynein [77]. Cycles of compression stabilise MTs in live cells [78,79] – albeit in *S. pombe*, acute compression in the cell ends triggers catastrophes [80]. Direct induction of catastrophe by mechanical compression is also seen *in vitro*. Tubulin is locked into the lattice to some extent – it takes appreciable force to remove subunits [81]. However, packing defects occur and can sensitise MTs to mechanical forces [82]. Interrogating the effects of mechanical forces at the lattice level on tubulin exchange and effector exchange poses appreciable challenges – yet methods are being developed because these effects are key to function.

## Discussion

Structural switching within the lattice, driven by allosteric effectors, is emerging as an important aspect of dynamic instability, enriching MT mechanobiology with an array of new structural and dynamic properties. Assembly into a MT not only stiffens PFs, it provides a new mechanical interface that allows neighbouring PFs to instruct one another, a structural memory, and a kind of calculator function, whereby the lattice integrates multiple inputs in order to remodel its own structure and function. The classical allosteric enzyme is haemoglobin [83], which has well-defined allosteric structural switching in response to many different effectors. However, haemoglobin is a tetramer. MTs too have a structural switch, but they also have thousands of subunits, can be built from multiple different isotypes, can be very extensively post-translationally modified, and can sense and exert mechanical force.

A critical unknown at present is the range of allosteric communication within the lattice [84]. Is mechanical signalling through the lattice limited to nearest neighbours, or does it go further [85]? Changing the PF number changes the structure of the growing tip and serves in this way as a kind of memory, since tip dynamics reflect this shift. But are there other memory effects, whereby conformational shifts that are reversible nonetheless persist for long periods? Many, perhaps most, MTs are mosaics of multiple tubulin isotypes. The mix affects the response, but we need to clarify whether isotypes freely mix or whether patches occur due to biased recruitment. Recombinant single isotype tubulins with defined properties [14] are powerful tools to address these questions, but getting definitive answers will also require that we live-track the distribution and conformation of tubulin molecules in the lattice with much-improved resolution. An important related challenge is to find ways to read out the PF numbers of different segments of dynamic MTs in the light microscope. It is especially important to understand the allosteric range in relation to drug action – how much of a particular effector needs to bind in order to switch the lattice conformation?

Less susceptible to wet experiments is the problem of quantifying local tension in the lattice. Here, computational simulation can be especially valuable to infer how mechanical forces impact lattice conformation and subunit exchange, and vice versa. Uniquely, computational simulation can provide insights into timescales and length-scales that are inaccessible to *ex silico* experiments [86,87]. As pointed out by one of our reviewers, modelling dynamic instability based on equilibrium data is appreciably more challenging than equilibrium modelling. Despite its challenges, computational simulation has greatly clarified thinking on the component reactions of dynamic instability [88]. Multiscale models, combining atomistic simulations with coarse-graining, can uncover how structural, mechanical [89] and chemical kinetic transitions are coupled [90]. Very recent work suggests that PF assembly and PF zipping indeed occur sequentially, with respect to particular PFs at a polymerising MT tip [91].

MTs are often described as scaffolding elements, but they are far more; they show subtle allostery [92], computing a structural response to a spectrum of lattice-binding effectors, thereby biasing the recruitment of further effectors and their own structural evolution. The same effector can have different effects on different tubulin isotypes in the lattice, variably influencing the ongoing recruitment of particular tubulins, MAPs, motors and lattice-binding drugs. PTMs [40,93,94] can directly affect tubulin–tubulin interactions and can steer, override or lock in effector recruitment [95,96] and related shifts in lattice conformation. Numerous questions need answers. How cooperative are lattice-conformational changes? Do clinically important [97] anti-MT drugs change the tubulin isotype composition of the lattice, and thereby their own effectiveness? Does conformational memory operate only by shifting PF number or can spacing changes and/or lattice curvature be metastably locked in, and if so, on what time scale? Recent advances in tubulin engineering [98], fluorophore engineering [99–101] and MT cryoEM [37] encourage us that definitive answers to these key questions are within reach.

**Perspectives**

- The GTP-coupled dynamic instability of MTs is fundamental to eukaryotic self-organisation. Recently, it has become clear that allosteric effectors other than GTP can induce structural switching deep within the subunit lattice of MTs, with broad consequences for MT biology.
- Dynamic MTs can respond to a battery of effectors, producing shifts in their lattice spacing and local curvature, introducing defects and/or changing the number of PFs in the lattice. Different tubulin isotypes can respond differently. These effects enrich our picture of dynamic instability, revealing the MT as a kind of mechanical calculator.
- Methods are emerging that can dissect allosteric structural switching in the MT lattice in molecular detail. Tubulin-directed allosteric drugs are critically important tools for medicine and agriculture. There is a pressing need to more fully to understand their actions.

## Abbreviations

EB: end-binding
GMPCPP: Guanosine-5'-[(β,γ)-methyleno]triphosphate
GMPPCP: Guanosine-5’-[(α,β)-methyleno]triphosphate
MAPs: MT-associated proteins
MT: microtubule
MTAs: MT-targeting agents
PF: protofilament
PTM: Post-Translational Modification
